# ENTPRISE: An Algorithm for Predicting Human Disease-Associated Amino Acid Substitutions from Sequence Entropy and Predicted Protein Structures

**DOI:** 10.1371/journal.pone.0150965

**Published:** 2016-03-16

**Authors:** Hongyi Zhou, Mu Gao, Jeffrey Skolnick

**Affiliations:** Center for the Study of Systems Biology, School of Biology, Georgia Institute of Technology, Atlanta, Georgia, United States of America; UMR-S665, INSERM, Université Paris Diderot, INTS, FRANCE

## Abstract

The advance of next-generation sequencing technologies has made exome sequencing rapid and relatively inexpensive. A major application of exome sequencing is the identification of genetic variations likely to cause Mendelian diseases. This requires processing large amounts of sequence information and therefore computational approaches that can accurately and efficiently identify the subset of disease-associated variations are needed. The accuracy and high false positive rates of existing computational tools leave much room for improvement. Here, we develop a boosted tree regression machine-learning approach to predict human disease-associated amino acid variations by utilizing a comprehensive combination of protein sequence and structure features. On comparing our method, ENTPRISE, to the state-of-the-art methods SIFT, PolyPhen-2, MUTATIONASSESSOR, MUTATIONTASTER, FATHMM, ENTPRISE exhibits significant improvement. In particular, on a testing dataset consisting of only proteins with balanced disease-associated and neutral variations defined as having the ratio of neutral/disease-associated variations between 0.3 and 3, the Mathews Correlation Coefficient by ENTPRISE is 0.493 as compared to 0.432 by PPH2-HumVar, 0.406 by SIFT, 0.403 by MUTATIONASSESSOR, 0.402 by PPH2-HumDiv, 0.305 by MUTATIONTASTER, and 0.181 by FATHMM. ENTPRISE is then applied to nucleic acid binding proteins in the human proteome. Disease-associated predictions are shown to be highly correlated with the number of protein-protein interactions. Both these predictions and the ENTPRISE server are freely available for academic users as a web service at http://cssb.biology.gatech.edu/entprise/.

## Introduction

Knowledge of an organism’s DNA sequence is not only indispensable for fundamental biological research, but is also of practical use in medical applications. With the rapid advance of next-generation sequencing technologies, it is now possible to obtain sequenced human exomes at affordable cost for use in personalized treatment and preventive diagnosis [1]. Since gene/protein variations are random and most variations have no harmful consequences [2], the detection of genetic variations is usually a necessary but not sufficient condition for the diagnosis of Mendelian disease. Thus, efficient and inexpensive methods that prioritize the identified variations for further experimental characterization are required. Many computational tools have been developed to address this need [3–14]. Some use only protein sequence information[3, 5, 7, 13], while others combine sequence with protein structural information[4, 6, 9, 14, 15]. There are also meta-methods that combine individual methods [16–19]. Most methods require training, e.g. PolyPhen-2[4], FATHMM[6], SNAP[8], and MUTPRED[9], while some do not, e.g. SIFT[3], and PANTHER[10]. Some methods can handle single nucleotide variations across the genome [14, 20] while others deal with amino acid variations within the exome [4, 12]. In this work, we focus on variations that cause protein amino acid changes.

Despite the good performance of each method as assessed by their respective authors, their accuracy has much room for improvement, especially for distinguishing disease-associated variations from neutral ones on the same protein/gene. For example, a recent study [17] shows that in a relatively rigorous test that separates testing data from training data, the best method, PhD-SNP[7] has a Mathews Correlation Coefficient (MCC) of 0.49 and an accuracy of 0.746. A consensus approach that combines these methods provides little improvement [17]. Ref. [17] is only relatively rigorous in that it has not tested the ability of discriminating variations on the same protein and the resulting effect on the false positive rate. Ref. [17]’s test results are clearly much worse than those of the authors for the same method. The reason for the performance discrepancy between the assessment of the authors and that of third parties [17, 21] is due to the overlap of variations between testing data and training data used by many methods [22]. Often they are trained and tested on datasets consisting of proteins having variations dominated either by neutral or disease-associated ones. This leads to the result that a method, which simply classifies a variation on the basis of whether it occurs in a protein implicated in disease or not, will perform well, independent of the nature of the variation itself. For example, FATHMM is optimized using disease-associated family/domain information and performs very well [6]. However, not until recently was its ability to discriminate disease-associated variations from neutral variations in the same gene/protein tested [22]. In fact, as we will show, FATHMM, which has very good performance on conventional test sets, i.e., the PredictSNP dataset and VariBench set [6, 17], gives the worst performance on a dataset consisting of proteins with balanced disease-associated and neutral variations. In practice, the discriminative ability to tell which variations in a disease-associated protein actually cause the disease or are neutral is as important as discriminating disease-associated proteins from non-disease-associated proteins. For example, not all variations in the BRCA1 and BRCA2 genes cause breast and/or ovarian cancer [2, 23]. In fact, the 1000 Genomes project found many neutral variations in disease-associated genes [2, 24]. Another issue with existing methods is that for neutral variations, they have not been tested on a large scale for false positives, e.g. variations from the 1000 Genomes project[2]. A method with a large false positive rate will identify far too many variations as disease-associated making the identification of true disease-associated variations difficult to find among the myriad of false positives, even though its recall rate is high.

In this work, we address these issues, especially the issue of discriminating disease-associated from neutral variations in the same protein/gene. We present a computational method that takes advantages of evolutionary conservation using protein sequence entropy and the local environment of a given variation using predicted protein three-dimensional (3D) structures. The combination of this information is realized through the boosted tree regression machine-learning algorithm[25, 26]. Sequence information representing evolutionary conservation was explored by almost all existing methods[3–9, 12]. As for protein structure information, in contrast to previous methods that are limited by available experimental structures[4] or predicted secondary structure and accessible surface area[9], we use the predicted three dimensional structures of all considered proteins. 3D structure allows us to derive features of the mutated position that encode possible domains and local environment information. In a recent comprehensive investigation[27], we found that disease-associated variations have structural properties different from neutral variations. Thus, we expect structure information will help discriminate disease-associated variations from neutral variations in the same protein.

In addition to testing on a conventional test dataset, to test the ability of methods to discriminate disease-associated variations from neutral variations on the same gene/protein, we construct a new balanced test dataset where each gene/protein has both neutral and disease-associated variations such that the ratio of neutral/disease-associated variations is between 0.3 and 3. This is in contrast to conventional test datasets (where most proteins have either solely neutral or solely disease-associated variations) that will favor methods classifying all variations in the selected protein as either all neutral or all disease-associated. In other words, a protein is classified as being either disease neutral or disease associated.

We have also carried out tests on large-scale neutral variations from the 1000 Genomes project[2] to test the false positive rates. Our approach, called **ENTPRISE**—a comprehensive combination of sequence **ENT**ropy and **PR**ed**I**cted protein **S**tructur**E** for predicting human disease-associated amino acid variations—shows consistent, significant improvement over widely used methods, e.g., SIFT[3], PolyPhen-2[4] for all tests.

We then apply ENTPRISE to predictions of disease-associated variations in nucleic acid binding proteins. Systematic analysis shows the interesting result that some proteins are “hot” in that most mutated positions are disease-associated while others are not. A possible explanation of “hot” proteins is the involvement of protein-protein interactions. The fraction of positions having disease-associated variations is highly correlated with number of protein-protein interactions the mutated protein experiences. It is also highly correlated with the probability of a protein being disease-associated. Thus, these predictions provide guidance for future experimental investigations.

## Materials and Methods

### Relative sequence entropy and sequence related features

Evolutionary sequence conservation information is utilized by all existing methods. Variations in a sequence conserved position are expected to be more prone to cause disease than non-conserved positions. Here, sequence conservation is represented by the relative sequence entropy defined as the difference of a given position’s entropy from the average entropy of all positions in a given protein:

RS=S−<S> (1)

where $S=-\sum_{l=1}^{20}f_{l}\ln(f_{l})$ is the sequence entropy of the given position, *f*_*l*_ is the normalized 3590 frequency profile. <> stands for averaging over the entire protein sequence.

The 3590 frequency profile is adopted from the PROSPECTOR threading algorithm for protein structure prediction [28], where 35 and 90 refer to two sequence identity cutoffs (see below). Specifically, a 3590 frequency profile is generated using a PSI-BLAST search [29] on the non-redundant sequence database downloaded from the Protein Data Bank (PDB) [30]. After each search, the sequences found by PSI-BLAST with E-value < 0.001 are filtered to have sequence identities between 35% and 90% to the query and each other. The reason for this filtering is to avoid over representation of certain sequences. These sequences and their alignments (by PSI-BLAST) are then used to jump start up to five iterations of a PSI-BLAST search. The final sequences and their alignments to the query sequence are used to derive the 3590 sequence frequency profile that counts the number of each amino acid type in each position of the query protein. The profile is then normalized by the number of sequences at each position. It should be noted that the 3590 profile does not bias towards any species or family of sequences: it could include sequences from human homologs/paralogs and orthologs from other closely related species. These sequences are assumed to be evolutionarily related. The entropy at a given position ranges from 0 to ln(20) = 2.996, with 0 being the most conserved and 2.996 the least. The relative entropy will remove some protein specific effects, such as when there are too few sequences for a profile calculation. For example, in the extreme case, if only the query sequence is used, every position will have a sequence entropy of zero, which corresponds to the lowest entropy value according to Eq 1.

The aforementioned relative entropy is one feature used in the boosted tree regression method[25, 26]. Other sequence-based features are variables encoding the wild amino acid type and variables for the mutated amino acid types. These features are based on the observation that certain types of variations are more frequent than others in disease associated variations[31]. Twenty variables are needed to encode the 20 types of amino acids. Each variable takes only two values: 1 if the amino acid it represents is present and 0 if not. Thus, 40 variables are needed to encode both wild type and mutant amino acid types.

### Structure prediction and structure related features

**Fig 1:**
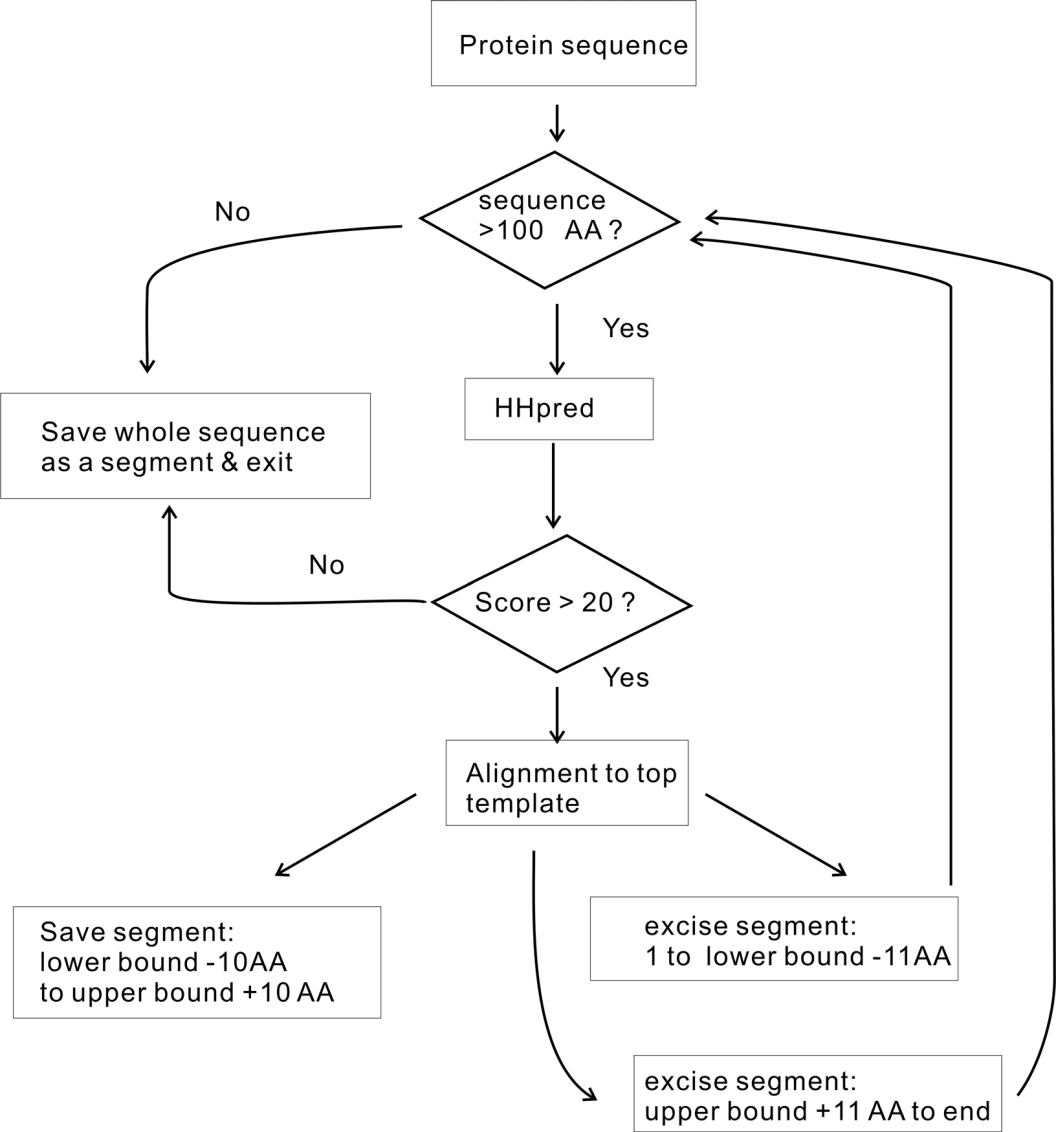
Protein sequence parsing procedure. The lower (upper) bound is defined as the aligned position of the template’s N-terminal (C-terminal) in the query sequence.

Protein structures were predicted by the latest variant of one of the best protein structure prediction methods TASSER^VMT^ [32–34]. To model long multi-domain proteins, we divide their sequences into smaller segments/domains (each segment itself could contain multiple domains) using the automated sequence parsing procedure shown in Fig 1 [35]. Specifically, HHPRED searches the query sequence against a representative experimental protein structure library, then the aligned query region in the top hit template is considered as a segment/domain of the query sequence. Since domains in our representative experimental protein structure library are based on SCOP domains[36], the partitioned domains are similar to those in SCOP rather than in PFAM[37]. After the query protein sequence is parsed, the structure of each segment/domain is independently modeled using TASSER^VMT^-lite[38]—a fast version of the TASSER^VMT^ method[34]. The top ranked model given by applying the SPICKER clustering method[39] to the low energy trajectories of the TASSER simulation[32] is the predicted structure for each segment/domain. We built structural models of the individual domains of 32,579 human protein targets. 85.6% (79.3%) have at least one domain whose predicted TM-score[38, 40] ≥ 0.4 (0.5) (a TM-score of 0.4 has a *p*-value of 3.4 x 10^−5^ [41]). The predicted TM-score was introduced in Ref[38] and is derived from TASSER simulations as a quality assessment of the predicted protein structures. Ref. [41] shows that protein pairs with a TM-score >0.5 are mostly in the same fold. In Ref [42], we found that the ligand homology based FINDSITE^comb^ for ligand virtual screening reaches its best performance as long as protein target model quality has a predicted TM-score ≥ 0.4. Thus a model with predicted TM-score ≥ 0.4 or 0.5 is not only statistically significant, but also practically meaningful. The list of predicted protein structures is available at http://cssb.biology.gatech.edu/repurpose/.

Structure related features that will be used for boosted tree regression[25, 26] consist of two classes: (1) segment/domain composition and (2) contacting residue composition. The segment/domain composition is described by the fractions of amino acid types in the whole segment/domain as found by HHPRED [35] based on its similarity and alignment to PDB templates (Fig 1). (This term is somewhat similar to the term in FATHMM related to families/domains [6]. FATHMM also uses a Hidden Markov Model (HMM) [35], as in HHPRED, to map the protein sequence to PFAM family/domains[43]). The domain composition provides a description of the whole domain. It can be used to distinguish proteins/domains where mutations are more likely to cause diseases [24]. Similarly, the contacting residue composition is represented by the fractions of amino acid types within 12 Å of the C_α_ distance from the mutated C_α_ position found in the predicted or experimental 3D structures. The 12 Å cutoff is adopted from a machine learning approach for predicting protein-ligand binding affinity[44] and is not optimized in this work; however, we will show that current approach is not sensitive to this cutoff. The contacting composition provides a description of the local pattern of the varied positions based on the observation that certain positions are more likely to cause diseases than others when mutated[31].

A summary of all features used in this work is given in Table 1. Except for the entropy and domain composition types that have been exploited by other methods, all other types of features in ENTPRISE are new.

**Table 1:** Summary of features.

Number of features	Description
1	Entropy derived from multiple sequence alignment
20	Wild-type amino acid type
20	Mutant amino acid type
20	Domain composition of 20 amino acid types
20	Contacting composition—composition of 20 amino acid types whose C_α_ atoms are within a 12 Å distance of the mutated C_α_ atom position

### Boosted tree regression for predicting disease-associated variations

Boosted tree regression has been employed in many applications[25, 26] and has been shown to be much better than support vector machines (SVM)[45] and random forests (RF)[46] in predicting genomic breeding values[47]. It involves generating a sequence of decision trees; each grows on the basis of the residuals of all previous trees[21, 26]. Here, a decision tree regression is implemented with a maximal depth of eight. The scoring function is represented as a boosted decision tree[26]:

$$f(x)=\sum_{m=1}^{N_{tree}}\varepsilon T_{m},$$ (2)

where *T*_*m*_ is a decision tree, *ε* is the shrinkage factor or learning rate, and *N*_*tree*_ is the number of trees. Two function values are adopted: 1 for disease-associated variations, and 0 for neutral variations. A total of 81 variables are used for regression: 1 from the relative entropy (Eq 1), 20 from the domain composition, 20 from the contacting residue composition, 20 encoding the wild type amino acid and 20 for the mutated amino acid type. The learning rate *ε* is set to 0.005, and the number of trees, *N*_*tree*_ is set to 2000. These values are purely empirical and not optimized.

### Alternative implementations of ENTPRISE

In order to tease out the effects of various factors affecting the overall performance of ENTPRISE, we implemented the following six alternative algorithms by changing or omitting one or few factors. **ENTPRISE_CUT8** changes the default C_α_ contact distance cutoff 12 Å to 8 Å; **ENTPRISE_NOTYP** does not use the 40 features encoding wild & mutant amino acid types; **ENTPRISE_NODOM** does not use the 20 domain composition features; **ENTPRISE_NOCNT** does not use the 20 contacting composition features; **ENTPRISE_NOENT** does not use the entropy feature; and finally **ENTPRISE_ONLYDOM,** that is very similar to FATHMM[6], uses only the 20 features of domain composition. A summary of all alternative implementations is given in Table 2.

**Table 2:** Summary of various implementations of ENTPRISE^a^.

Method	Feature type			
	amino acid type	domain composition	contacting composition (default cutoff = 12Å)	entropy
ENTPRISE	√	√	√	√
ENTPRISE_CUT8	√	√	√ (cutoff = 8Å)	√
ENTPRISE_NOTYP		√	√	√
ENTPRISE_NODOM	√		√	√
ENTPRISE_NOCNT	√	√		√
ENTPRISE_NOENT	√	√	√	
ENTPRISE_ONLYDOM		√		

^a^ Symbol √ means feature type is used.

### Data sets

#### ENTPRISE-TR & ENTPRISE-TE sets

For training and test our regression model, we utilize the datasets from the PredictSNP method [17]. We combine both the training set **(**called **OVERFIT** in Ref. [17]) and test set (called **PredictSNP** in Ref. [17]) that are collected from the training sets of a number of methods including PolyPhen-2 [4] trained on the 41,918 variations of HumVar data from SwissProt [48]. We map the proteins to our human proteins having predicted three-dimensional structures. This dataset is highly biased in that many proteins with disease-associated variations have no or very little neutral variation data (only 639 of 1,782 proteins have a ratio of neutral/disease-associated variations > 0.3). Likewise, most proteins containing neutral variations do not have any disease-associated variations (11,120 of 12,902 proteins). A method trained on this kind of set can perform very well simply by classifying proteins and predicting every variation as being disease-associated in case of disease-associated proteins, and as neutral in the case of neutral proteins. To alleviate this biasing issue in training, we enrich this dataset by adding neutral variations identified by the 1000 Genomes project consortium, which has sequenced healthy individuals. The assumption is that most variations in the 1000 Genomes project are likely to be neutral [2]. In fact, as we estimate in the Results section, its false negative rate is < 2%. For each protein with disease-associated variations, we add all neutral, nonsynonymous variations of the same gene from the 1000 Genomes project. After removing redundantly added variations (when > = 2 isoforms of a protein exist, duplicated variations can be added), we obtained a set of 12,902 proteins with 30,569 disease-associated & 69,296 neutral variations. In this enriched set, 1,522 of the 1,782 proteins having disease-associated variations have the ratio neutral/disease-associated > 0.3. Thus, for proteins with disease-associated variations, our enriched set is much more balanced than the data from the PredictSNP method. A method that simply classifies all variations on proteins having disease-associated variations to be disease-associated will receive no advantage in performance.

We then randomly partition the above set at the variation level into two approximately equal-sized sets called **ENTPRISE-TR** and **ENTPRISE-TE** sets for training and testing, respectively. **ENTPRISE-TR** has 9,728 proteins with 14,497 disease-associated variations on 1,458 proteins & 32,163 neutral variations on 9,618 proteins, of which 22,424 variations are on 8,270 proteins without any disease-associated variations. **ENTPRISE-TE** has 9,725 proteins with 14,443 disease-associated variations on 1,461 proteins & 32,131 neutral variations on 9,609 proteins, of which 20,669 variations are on 8,264 proteins without any disease-associated variations.

**ENTPRISE-balance set. ENTPRISE-balance** is not an independent set and is a subset of the above **ENTPRISE-TE** set having balanced disease-associated and neutral variations on the same protein. In this set, the ratio of neutral/disease-associated variations of proteins having disease-associated variations is between 0.3 and 3.0. It has 761 proteins with 4,501 disease-associated and 4,406 neutral variations. **ENTPRISE-balance** was created for testing methods’ performance on discriminating variations in the same protein. All three data sets along with their associated protein sequences are available at http://cssb.biology.gatech.edu/entprise/.

A summary of the above three sets as well as the duplicate-sampled ENTPRISE-TR described below in training protocol is given in Table 3.

**Table 3:** Summary of variations in ENTPRISE datasets.

Set	Disease-associated			Neutral			Total
	In balanced protein^a^	In unbalanced protein^a^	Sub-total	In balanced protein	In unbalanced protein	Sub-total	
ENTPRISE-TR	4,727 (751)	9,770 (707)	14,497	4,489 (751)	27,674 (8,866)	32,163	46,659 (9,728)
ENTPRISE-TE	4,501 (761)	9,942 (700)	14,443	4,406 (761)	27,725 (8,847)	32,131	46,574 (9,725)
ENTPRISE-balance	4,501 (761)	-	4,501	4,406 (761)	-	4,406	8,907 (761)
Duplicate sampled ENTPRISE-TR	28,362	9,770	38,132	26,934	27,673	54,607	92,739

^a^ Balanced protein is defined as a protein whose ratio of neutral/disease-associated variations is between 0.3 and 3.0. Unbalanced protein has its ratio out of the above range. Numbers in parenthesis are numbers of proteins.

#### 1k-Genome & VariSNP sets

For the false positive test, a large set selected from the 1000 Genomes data (~1/3) that have no overlap with the training data is employed. This set called the **1k-Genome** set consists of 156,799 variations from 17,298 proteins. Of these, 60,026 variations (38.3%) are from 7,039 (40.7%) proteins known to be disease-associated according to the GeneCards database (www.genecards.org) [24]. While there is no guarantee that 100% variations of the **1k-Genome** set are neutral variations, we assume that its false negative rate is negligibly small. In practice, in the Results section, we estimate its false positive rate to be <2% and note that no current method can achieve this level. To further confirm our assumption, we also test methods on the expert filtered neutral variation set **VariSNP** [49].

The wild type Human protein sequences are downloaded from ftp://ftp.ncbi.nih.gov/genomes/H_sapiens/protein/[49].

### ENTPRISE training protocol

Our training set, ENTPRISE-TR represents the natural distribution of real world observed data. However, as shown in Table 3, it is a highly imbalanced data because out of the 32,163 neutral variations, only 4,489 (1/7) are in the 751 proteins with balanced neutral/disease-associated variations (ratio neutral/disease-associated is between 0.3 and 3). Machine learning approaches usually bias towards the majority class (here, bias towards neutral variations on proteins having very few or no disease-associated variations) [50]. Thus, patterns of disease-associated variations in proteins with balanced variations will not be learned if the method is trained naively on ENTPRISE-TR set. Investigation has shown that for a tree-based approach as in this work[51], training on the naturally occurring distribution (even though biased) generally gives optimal performance if evaluated using classification accuracy, whereas training on a balanced distribution performs better if evaluated using area under the ROC curve (AUC). Since use of accuracy is heavily biased to favor the majority class (in reality, the neutral variations on proteins having no disease-associated variations overwhelmingly dominate), we shall use AUC criteria for training on the balanced distribution.

There are two basic ways of reducing imbalance in training data: under-sampling and over-sampling [51]. Under-sampling uses only a subset of the majority class and thus could throw away useful data. Over-sampling replicates examples of the minority class and thus increases sample size and training cost. In this work, we employ over-sampling by replicating by 5 times those variations (for a total of 6 copies) on the 751 proteins having the ratio of neutral/disease-associated between 0.3 and 3. The final samples used for training have approximately equal numbers of neutral variations in proteins having balanced neutral/disease-associated variations and in other proteins (proteins having the ratio neutral/disease-associated < 0.3 or > 3; see Table 3 columns 5 & 6, bottom row).

### Evaluation

For ENTPRISE-TE and ENTPRISE-balance benchmarking data sets, we evaluate methods using the following criteria:

$$\text{Matthews correlation coefficient}\;(\text{MCC})=\frac{tp\;*\;tn-fp\;*\;fn}{\sqrt{\left(tp+fp)\;*\;(tp+fn)\;*\;(tn+fp)\;*\;(tn+fn\right)}}$$ (3)

$$\text{Accuracy}\;(\text{ACC})=\frac{tp+tn}{tp+tn+fp+fn}$$ (4)

$$\text{Sensitivity}\;(\text{Sen})=\frac{tp}{tp+fn}$$ (5)

$$\text{Specificity}\;(\text{Spe})=\frac{tn}{tn+fp}$$ (6)

Positive predictive valuePPV=tp/(tp+fp) (7)

Negative predictive valueNPV=tn/(tn+fn) (8)

and the area under the receiver operating characteristic curve (AUC). *tp*, *tn*, *fp*, *fn* are the numbers of true positives, true negatives, false positives and false negatives for disease-associated variations, respectively.

### Application of ENTPRISE to human transcription machinery

A list of experimentally verified or putative nucleic acid binding proteins in the human proteome was obtained from UNIPROT (1/7/2015 release) [52]. A total of 3,056 proteins ≥ 40 residues in length were mapped to our human genome proteins having predicted protein structures. About 71% of these are DNA-binding and 22% are RNA-binding proteins. Most have an annotated GO molecular function related to transcription [15]. For completeness, at each residue position, the ENTPRISE scores for all 19 types of amino acid variations other than the reference amino acid itself are calculated. Of course, most of these variations are less likely because they require that more than one nucleotide be varied. A position is defined as a “hot” spot if all amino acid variations lead to an ENTPRISE score > 0.45. In verifying whether a protein is disease-associated or not, we used GeneCard data [24]. The number of protein-protein interactions of a protein was obtained from the HIPPIE database[27].

## Results

### Comparison to other methods

The results on the test set ENTPRISE-TE are shown in Table 4 where we compare ENTPRISE to the widely used methods SIFT [12] and PolyPhen-2 (PPH2) [4] and the well performing methods MUTATIONASSESSOR [13, 53], MUTATIONTASTER [14], and FATHMM [6]. Performance of alternative implementations of ENTPRISE are also shown to examine the effect of various features/factors. Although there are other outstanding methods such as CADD [15] and MUTATIONTASTER2 [14], we are not able to compare our method with them due to the fact that they require DNA variation as input. Nevertheless, we note that MUTATIONTASTER2 has very little improvement over MUTATIONTASTER for single amino acid variation data[14]. All results for other methods than ENTPRISE were extracted from the dbNSFP database version 2.9 downloaded from https://sites.google.com/site/jpopgen/dbNSFP [54]. To be fair to other methods, only their overlapping predictions with ENTPRISE are evaluated. In addition to be slightly advantageous to them, possible overlapped variations of their training data with ENTPRISE-TE test set are not excluded since not all methods have their training data available. Performance is evaluated using the Matthews correlation coefficient (MCC), accuracy, sensitivity, specificity, PPV, NPV and AUC. The number of evaluated variations is also included. The optimal cutoff for ENTPRISE is 0.45, which was obtained by optimizing the MCC value using the training ENTPRISE-TR set. In practice, we can increase the cutoff to lower false positive rate, or decrease the cutoff to increase recall rate. Therefore, we also present ENTPRISE with two additional score cutoffs 0.2 & 0.55 for reference when a greater recall rate or smaller false positive rate is preferred.

**Table 4:** Performance of different methods on the ENTPRISE-TE set.

Method	Evaluated variations^a^	MCC	ACC	Sen	Spe	PPV	NPV	AUC
SIFT	40,120	0.395	0.674	0.815	0.598	0.520	0.858	0.786
PPH2-HumDiv	40,317	0.374	0.646	0.846	0.539	0.496	0.867	0.771
PPH2-HumVar	40,317	0.423	0.700	0.796	0.648	0.548	0.855	0.793
MUTATIONASSESSOR	39,758	0.417	0.710	0.744	0.692	0.564	0.834	0.788
MUTATIONTASTER	40,286	0.337	0.600	**0.886**	0.447	0.462	0.879	0.695
FATHMM	39,741	0.538	0.764	0.838	0.724	0.623	0.891	0.866
ENTPRISE(0.45)^b^	46,574	**0.645**	**0.847**	0.768	**0.883**	**0.746**	**0.894**	**0.907**
ENTPRISE(0.55)^b^	46,574	0.646	0.854	0.668	0.937	0.827	0.863	0.907
ENTPRISE(0.20)^b^	46,574	0.488	0.688	0.947	0.572	0.499	0.960	0.907
ENTPRISE_CUT8	46,574	0.651	0.850	0.764	0.889	0.755	0.893	0.908
ENTPRISE_NOTYP	46,574	0.645	0.850	0.738	0.900	0.768	0.884	0.906
ENTPRISE_NODOM	46,574	0.464	0.763	0.674	0.803	0.606	0.846	0.812
ENTPRISE_NOCNT	46,574	0.680	0.865	0.747	0.918	0.805	0.890	0.919
ENTPRISE_NOENT	46,574	0.645	0.851	0.716	0.912	0.786	0.877	0.903
ENTPRISE_ONLYDOM	46,574	0.691	0.860	0.855	0.862	0.736	0.930	0.913

^a^ To be fair to other methods, only their overlapped variations with those of ENTPRISE are evaluated.

^b^ ENTPRISE with alternative score cutoffs 0.20, 0.45(default), 0.55.

Table 4 shows that ENTPRISE outperforms all other methods significantly with a MCC of 0.645 compared to the next best method FATHMM with a MCC of 0.538. Except for a sensitivity of 77%, ENTPRISE has the best values for all other measures. PPH2 has the best coverage of all methods given the same number of input variations overlapped with ENTPRISE. MUTATIONTASTER has the best sensitivity (0.89) while having the worst specificity (0.447) and overall performance based on MCC (0.337) & AUC (0.695) due to its over-predictions for disease-associated variations.

By examining the performance of various alternative implementations of ENTPRISE, we find that ENTPRISE is not sensitive to C_α_ distance cutoff for defining the contacting composition, as ENTPRISE_CUT8 is almost indistinguishable from ENTPRISE. The most important feature is the domain composition. Without it (ENTPRISE_NODOM), ENTPRISE will have MCC of 0.464 (though it is still better than many other methods). Using the domain composition features alone (ENTPRISE_ONLYDOM, equivalent to FATHMM) will result in MCC of 0.691 that is even better than that of full ENTPRISE. However, it will have very poor discrimination ability for variations on the same protein as tested next on the ENTPRISE-balance set. Similar considerations remove ENTPRISE_NOCNT as the method of choice. Other features seem to have very little effect on this test set.

**Fig 2:**
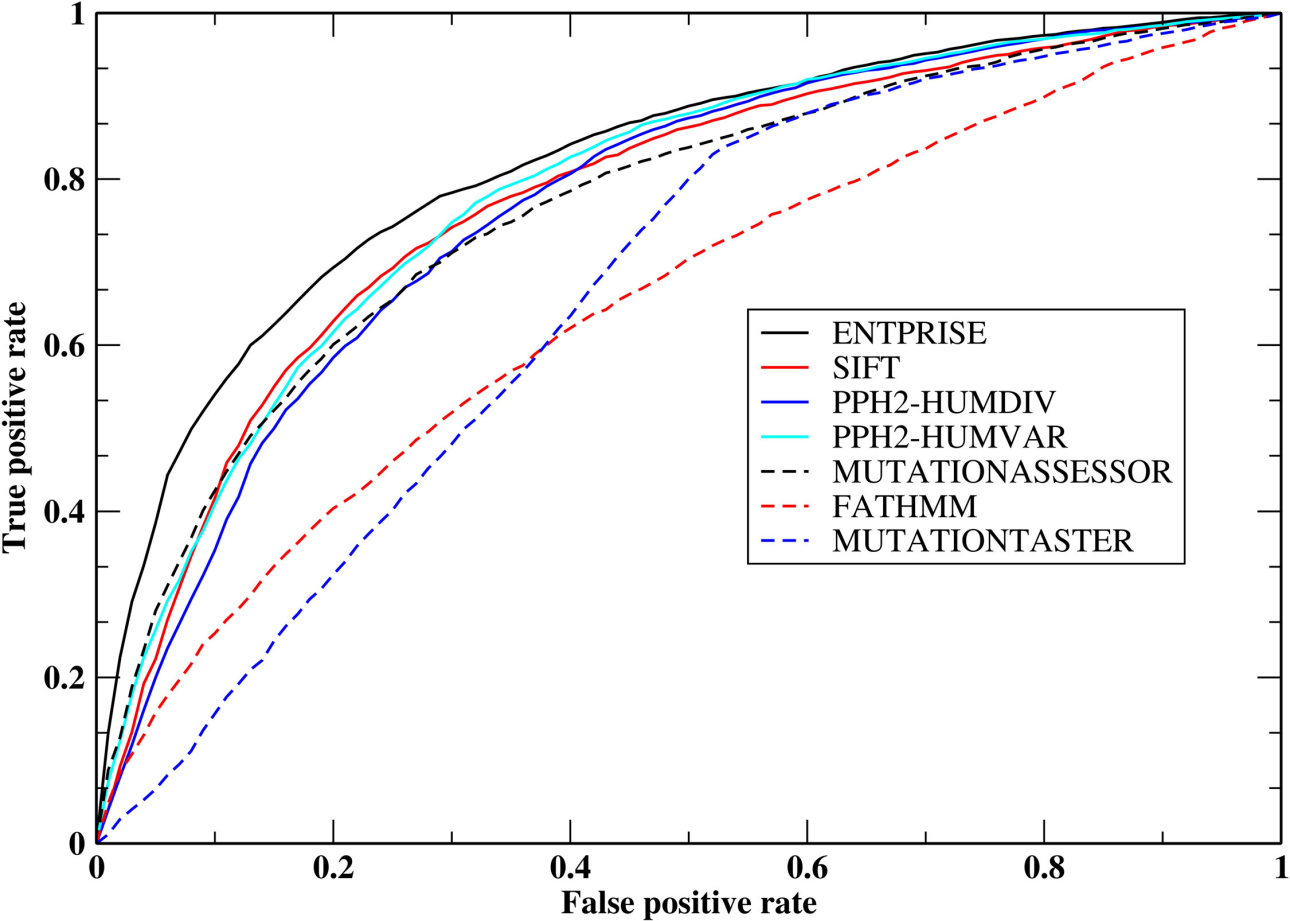
Receiver operating characteristic curves of methods ENTPRISE, SIFT, PP2-HUMDIV, PPH2-HUMVAR, MUTATIONASSESSOR, MUTATIONTASTER & FATHMM for the ENTPRISE-balance set.

Table 5 presents the performance of the various methods on the ENTPRISE-balance set. Again, except for ENTPRISE, all results are extracted from the dbNSFP database, and to be fair to other methods, only their overlapping predictions with ENTPRISE are evaluated. A significant performance change is observed for ENTPRISE_ONLYDOM and FATHMM. Now, FATHMM (ENTPRISE_ONLYDOM) performs the (2nd) worst, whereas ENTPRISE is still the top performer based on MCC or AUC followed by PPH2-HumVar. This demonstrates that ENTPRISE is not biased towards separating disease-associated proteins from neutral proteins as opposed to FATHMM & ENTPRISE_ONLYDOM, and is much better than other methods in discriminating disease-associated variations from neutral ones on the same gene/protein as measured by MCC; this is a key advantage. The ROC curves of the compared methods for the ENTPRISE-balance set are shown in Fig 2. ENTPRISE has the best true positive rate at all false positive rate levels. At a 10% false positive rate, ENTPRISE has a true positive rate of ~54%; whereas the next best method, MUTATIONASSESSOR has a true positive rate of ~ 42%.

**Table 5:** Performance of different methods on the ENTPRISE-balance set.

Method	Evaluated variations^a^	MCC	ACC	Sen	Spe	PPV	NPV	AUC
SIFT	8,438	0.406	0.699	0.826	0.565	0.668	0.754	0.775
PPH2-HumDiv	8,522	0.402	0.693	0.863	0.511	0.652	**0.778**	0.771
PPH2-HumVar	8,522	0.432	0.714	0.807	0.616	0.691	0.750	0.786
MUTATIONASSESSOR	8,455	0.403	0.702	0.748	0.653	0.696	0.709	0.765
MUTATIONTASTER	8,517	0.305	0.638	**0.886**	0.375	0.601	0.756	0.662
FATHMM	8,459	0.181	0.592	0.693	0.484	0.588	0.598	0.637
ENTPRISE(0.45)^b^	8,907	**0.493**	**0.742**	0.669	**0.819**	**0.790**	0.708	**0.818**
ENTPRISE(0.55)^b^	8,907	0.472	0.721	0.551	0.893	0.841	0.661	0.818
ENTPRISE(0.20)^b^	8,907	0.394	0.677	0.906	0.443	0.624	0.821	0.818
ENTPRISE_CUT8	8,907	0.493	0.742	0.660	0.827	0.795	0.704	0.815
ENTPRISE_NOTYP	8,907	0.437	0.713	0.611	0.817	0.773	0.673	0.788
ENTPRISE_NODOM	8,907	0.453	0.725	0.678	0.773	0.753	0.702	0.792
ENTPRISE_NOCNT	8,907	0.469	0.727	0.607	0.848	0.803	0.679	0.811
ENTPRISE_NOENT	8,907	0.401	0.691	0.554	0.832	0.771	0.646	0.779
ENTPRISE_ONLYDOM	8,907	0.254	0.626	0.709	0.542	0.612	0.645	0.670

^a^ To be fair to other methods, only their overlapped variations with those of ENTPRISE are evaluated.

^b^ ENTPRISE with alternative score cutoffs 0.20,0.45(default),0.55.

Table 5 also shows that the most important feature contributing to ENTPRISE’s discriminating ability is the entropy. Without it (ENTPRISE_NOENT), its MCC decreases from 0.493 to 0.401. Other features ordered by their importance are: amino acid type, domain composition, and contacting composition. Amino acid type features contribute to ENTPRISE’s discrimination ability is due to that certain type of variations (e.g. variations disrupt or form disulfide bonds, secondary structures) are more prevalent in disease-associated variations than in neutral variations as found by our recent work [31]. A notable result in Table 5 is that domain composition also has a contribution to discrimination ability. Without it, ENTPRISE’s MCC decreases from 0.493 to 0.453. Even though domain composition has no position specific information, it could serve as a modulator for other position specific features. For example, a variation on a protein surface is usually harmless, but if the protein has a binding partner and that variation is near the binding site, it could be harmful[31].

Another challenging test that so far has not been performed by existing methods is on a large set comprised of entirely neutral variations, e.g., those from the 1000 Genomes project [2]. Even though these variations might not be 100% neutral, we can fairly assume that the false negative or true positive rate is negligibly small. When there is a large number of variations found in a given genome, a large false positive rate makes it very difficult to identify true positive targets for further experimental characterization or possible treatment. Thus, it is important to have this kind of benchmarking test. The result for the test on the 1000 Genomes set along with VariSNP is shown in Table 6. Again, all results of other methods other than ENTPRISE are extracted from the dbNSFP database. Obviously, ENTPRISE has a significantly lower false positive rate of 10.7%. FATHMM comes in second at 12.9%. The false positive rates of the other methods are very high: 42.6% by SIFT, 48.7% by PPH2-HumDiv, 36.4% by PPH2-HumVar, 29.3% by MUTATIONASSESSOR, and 51.5% by MUTATIONTASTER. Tests on the expert filtered VariSNP dataset show an identical trend. The maximal difference between tests on the 1000 Genomes and VariSNP database is only 5%. All methods but the biased ENTPRISE_ONLYDOM & FATHMM have slightly lower false positive rates.

**Table 6:** Performance of different methods on the 1000 Genome & VariSNP sets.

	1k-Genome		VariSNP	
Method	Evaluated variations^a^	False positive rate	Evaluated variations^a^	False positive rate
SIFT	151,182	42.6%	70,430	38.7%
PPH2-HumDiv	151,981	48.2%	70,758	44.2%
PPH2-HumVar	151,981	36.4%	70,758	31.6%
MUTATIONASSESSOR	149,248	29.3%	69,520	26.1%
MUTATIONTASTER	151,830	51.5%	70,645	46.4%
FATHMM	143,032	12.9%	67,433	14.2%
ENTPRISE(0.45)^c^	162,249	**10.7%**	61,215	**9.0%**
ENTPRISE(0.55)^c^	162,249	5.4%	61,215	4.4%
ENTPRISE(0.20)^c^	162,249	42.0%	61,215	39.4%
ENTPRISE_CUT8	162,249	10.1%	61,215	8.5%
ENTPRISE_NOTYP	162,249	8.1%	61,215	6.9%
ENTPRISE_NODOM	162,249	20.5%	61,215	18.5%
ENTPRISE_NOCNT	162,249	6.4%	61,215	5.3%
ENTPRISE_NOENT	162,249	6.7%	61,215	5.7%
ENTPRISE_ONLYDOM	162,249	2.6%	61,215	3.0%

^a^ To be fair to other methods, only overlapping variations with ENTPRISE are evaluated.

^b^ Number of input variations: 74,837.

^c^ ENTPRISE with alternative score cutoffs 0.20,0.45(default),0.55.

The false positive rates (FPR) of six methods (excluded FATHMM & all alternatives of ENTPRISE) on the two sets have a Pearson’s correlation coefficient of 0.999 with the fitting: FPR(1kgenome) = FPR(varisnp)*1.075+0.015±0.005. A perfect method should have FPR(varisnp) = 0 and will have FPR(1kgenome) = 0.015±0.005. This extrapolated value corresponds to the apparent true positive or false negative rate of the 1k-Genome set, which is < 2%.

By examining the performance of alternative ENTPRISE methods and FATHMM on this set, we find that the domain composition has the most important contribution to lowering the false positive rate of ENTPRISE. Thus, domain composition has a favorable contribution to all three test sets (ENTPRISE-TE, ENTPRISE-balance & 1k-Genome/VariSNP).

A ten fold cross-validation of ENTPRISE at PFAM[43] family level can be found in the S2 File which shows consistent good performance of ENTPRISE (MCC = 0.571) in the possible hardest condition that the variations are on proteins having no common family/domain with the training set.

To test how robust ENTPRISE is to varying fraction of disease-associated variations used in the learning procedure, we carried out a new training procedure that uses only observed variations. This training set differs from our standard training set, i.e., the balanced set, where duplicated variations are introduced to balance the proteins having both disease-associated variations and neutral variations in the training data (otherwise many proteins contain only disease-associated or only neutral variations). We trained ENTPRISE on observed variation data (i.e. ENTPRISE-TR without duplicating variations on proteins in the training set). In practice, this set mainly consists of proteins with only neutral variations. In S2 File and S4 Table, we present the performance of ENTPRISE trained on observed variation data. By examining S4 Table that compares performance for two learning procedures, we found that the difference is not too large; ENTPRISE still performs better than other methods that trained on the observed data. For the test on the usually used kind of data ENTPRISE-TE, ENTPRISE is even slightly better. For testing on our new, balanced test data, ENTPRISE-balance, ENTPRISE is only slightly worse. Thus, ENTPRISE is robust to the varying fraction of disease-associated variants used in the learning procedure.

### Performance of ENTPRISE for predicting cancer driver missense mutations

We perform a test of ENTPRISE for predicting cancer driver missense mutations using the benchmark sets from Ref.[53]. The test results are compiled in Tables 7, 8 and 9. The coverages of ENTPRISE for COSMIC, TCGA, COBR sets are 2,263/2,682(84.4%), 354/455(77.8%), 105/147(71.4%), respectively. For the COSMIC set, with an accuracy of 85.3%, ENTPRISE is second only to the cancer-specific predictor CHASM[55] that was trained on COSMIC data and has accuracy of 89%. For this set, ENTPRISE is better than MutationAssessor with its 81% accuracy and PolyPhen-2 with its 71% accuracy[53]. However, like CHASM, the performance of ENTPRISE on the TCGA and COBR sets drops considerably. For the TCGA set, ENTPRISE is only slightly better than CHASM and the logRE methods, and for the COBR set, it is just slightly better than the CHASM, logRE and mCluster methods. The reason for the good performance of ENTPRISE for the COSMIC set could be due to the possibility that many cancer driver mutations behave the same way as mutations causing Mendelian diseases that ENTPRISE was trained upon. The reason for the drop in performance of ENTPRISE as well CHASM on the TCGA and COBR sets could be attributed to the fact that both ENTPRISE and CHASM are machine learning methods that require a set of common features between training data and testing data. It was noted that mutations matching to COSMIC variants were excluded from the TCGA and COBR sets and those excluded mutations were the ones with highest frequencies in these two test sets. Thus, there is a drop in severity of mutations in the latter two test sets causing the performance difference[53](i.e. the latter two sets have weaker disease-causing signal than the COSMIC set).The good performance of ENTPRISE on the COSMIC set demonstrates that ENTPRISE is a good choice for annotating somatic as well as germline mutations.

**Table 7:** Performance of ENTPRISE for predicting cancer driver missense mutations in the COSMIC set.

Method^a^	MCC	ACC	Sen	Spe	AUC	False positive rate = 1-Spe
ENTPRISE	0.724	0.853	0.745	0.961	0.914	3.9%
CHASM	0.79	0.89	0.79	0.99	0.92	1.0%
MUTATIONASSESSOR	0.62	0.81	0.76	0.86	0.89	14%
Condel	0.58	0.78	0.75	0.82	0.85	18%-
PolyPhen-2	0.54	0.77	0.79	0.75	0.82	25%
SIFT	0.52	0.76	0.70	0.82	0.80	18%
SNAP	0.37	0.68	0.55	0.81	0.67	19%
mCluster	0.35	0.65	0.40	0.90	0.64	10%
logRE	0.22	0.61	0.65	0.57	0.60	43%

^a^ Results of other methods are taken from Ref.[53] Table 1.

**Table 8:** Performance of ENTPRISE for predicting cancer driver missense mutations in the TCGA set.

Method^a^	MCC	ACC	Sen	Spe	AUC	False positive rate = 1-Spe
ENTPRISE	0.098	0.530	0.133	0.927	0.588	7.3%
CHASM	0.05	0.50	0.0	1.00	0.34	0%
MUTATIONASSESSOR	0.49	0.74	0.86	0.62	0.79	38%
Condel	0.37	0.68	0.66	0.66	0.72	34%
PolyPhen-2	0.34	0.66	0.76	0.56	0.68	44%
SIFT	0.30	0.65	0.74	0.56	0.66	44%
SNAP	0.26	0.62	0.43	0.79	0.59	21%
mCluster	0.17	0.54	0.08	0.99	0.50	1%
logRE	0.07	0.52	0.39	0.64	0.50	36%

^a^ Results of other methods are taken from Ref.[53] Table 2.

**Table 9:** Performance of ENTPRISE for predicting cancer driver missense mutations in the COBR set.

Method^a^	MCC	ACC	Sen	Spe	AUC	False positive rate = 1-Spe
ENTPRISE	0.198	0.562	0.171	0.952	0.585	4.8%
CHASM	0.08	0.50	0.0	1.0	0.36	0%
MUTATIONASSESSOR	0.46	0.70	0.91	0.50	0.74	50%
Condel	0.33	0.66	0.66	0.66	0.68	34%
PolyPhen-2	0.30	0.65	0.63	0.66	0.63	34%
SIFT	0.29	0.64	0.73	0.55	0.63	45%
SNAP	0.26	0.62	0.45	0.78	0.59	22%
mCluster	0.0	0.50	0.0	1.0	0.46	0%
logRE	0.08	0.54	0.44	0.64	0.53	36%

^a^ Results of other methods are taken from Ref.[53] Table 3.

### Application of ENTPRISE to human transcription machinery

**Fig 3:**
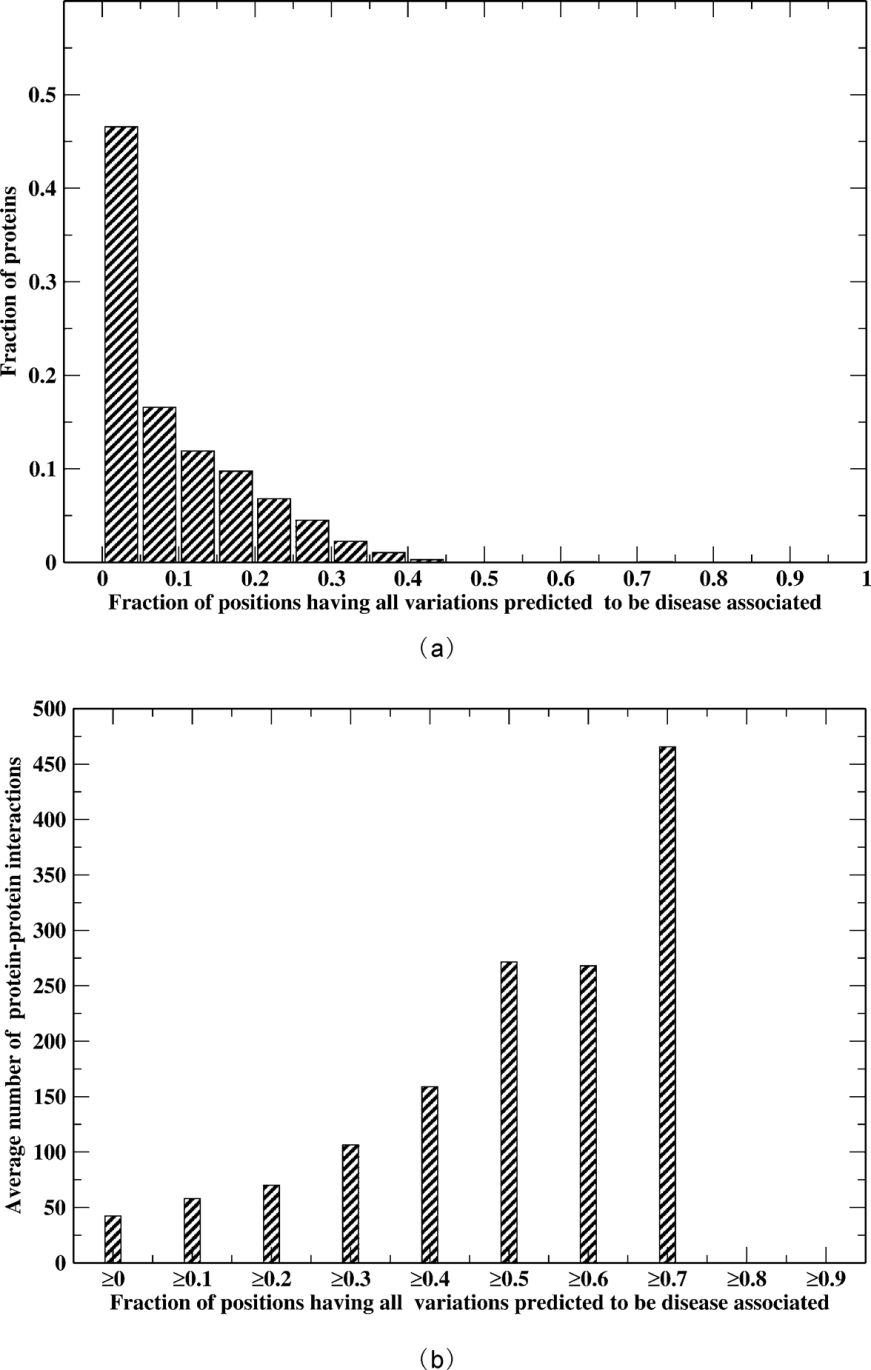
(a) Normalized distribution of proteins versus the fraction of positions having all variations predicted to be disease-associated; (b) Average number of protein-protein interactions versus the fraction of positions having all variations predicted to be disease-associated ≥ the given threshold value.

We applied ENTPRISE to 3,041 nucleic acid binding proteins, most of which are DNA or RNA binding proteins involved in transcription. We analyze their “hot” spots defined as positions predicted to be disease-associated for all possible variations regardless of variation type. The normalized distribution of proteins versus the fraction of “hot” spots is shown in Fig 3(A). The fraction is the average of all isoforms of a given protein. Around 47% of proteins have a “hot” spot fraction < 5%. Only seven proteins have a hot spot fraction > 50%. The “hottest” protein is TP53, where 74% of its positions are “hot”. It is well-known that variations of TP53 are the cause of many cancers. In fact, 255 positions of its total of 393 residues likely have cancer driver variations that are defined as variations having occurrences > = 2 samples in the COSMIC database (v66) [56]. Finding the driver variations in cancers is a hard and unsolved problem. Currently, the best computational option is frequency-based and has been employed for assessing the performance of computational methods for predicting such variations [53, 57]. For this particular protein, there are far more training deleterious variations (1,653 covering 345 residues) than others (the next number of training deleterious variations is 220 for the AR gene). Thus, our predictions for this protein are consistent with experimental data. Our predictions are also consistent with the predictions of other methods. For example, the predicted fraction of positions having pathogenic variations is 88% by SIFT, and 83% by PPH2_HumDiv. However, the large fraction of “hot” spots could also be due to the bias of experimental data itself (far more disease-associated variations than neutral ones: 1653 vs. 7 in our training data). The second “hottest” protein is encoded by the HNF1A gene that has 73% of its positions as “hot” spots. However, there are only 151 deleterious variations covering 128 or 20% of the total 631 residues of the protein in the training dataset. Therefore, our predictions do not just memorize the training data. HNF1A (homeobox A) is known to be associated with nineteen types of disorders [24].

To understand why some genes like TP53 are so fragile to variations (viz. most of their positions are “hot”), we hypothesize that these proteins have, on average, more protein-protein interactions than others so that more of their surface residues are involved; in addition, buried residues are important for monomer stability[31]. There are protein-protein interaction data for 2,629 of the 3,056 proteins in the HIPPIE database[27]. Fig 3(B) shows the average number of interactions of proteins having a fraction of “hot” spots equal to or greater than given threshold. Obviously, the average number of interactions is highly correlated with the hot spot fraction; the Pearson’s Correlation Coefficient is 0.93. The “hottest” protein TP53 interacts with 877 proteins and is responsible for the large peak in the far right of Fig 3(B). Thus, disruption of protein-protein interactions is likely the cause of a surface position being disease-associated when it is mutated.

**Fig 4:**
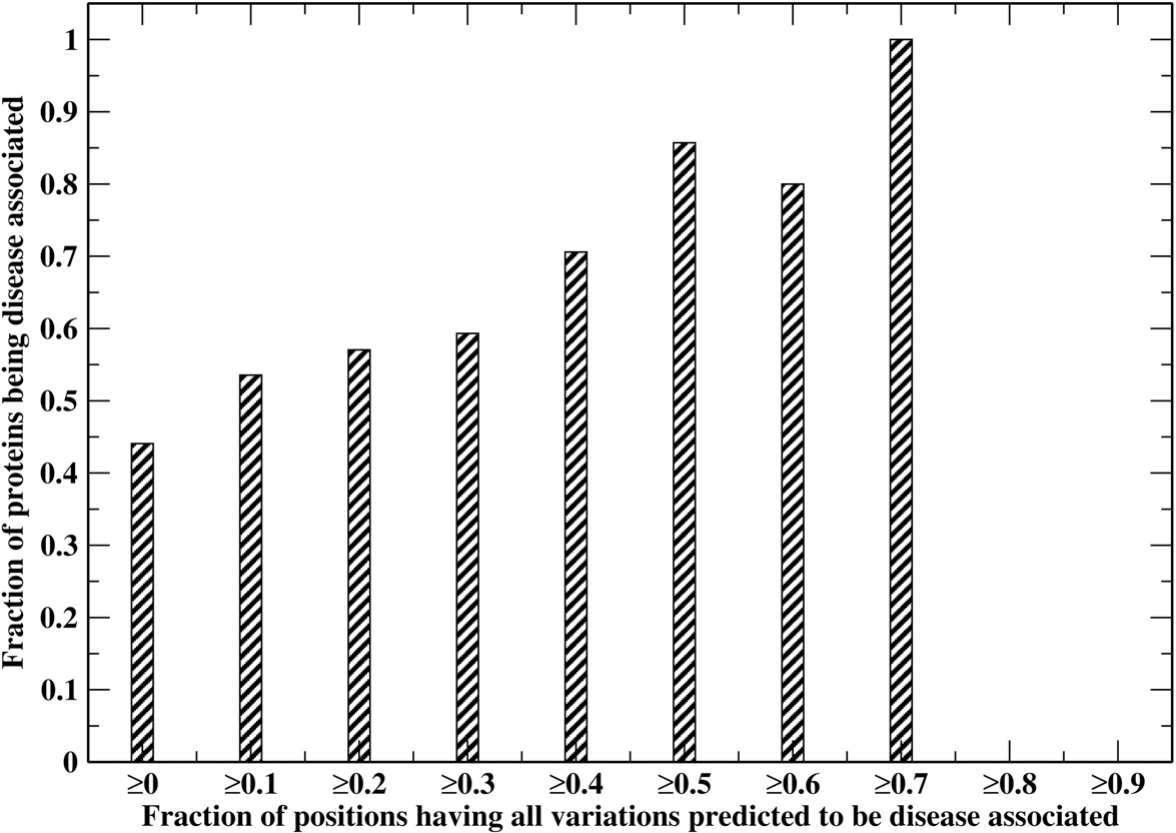
Cumulative fraction of proteins predicted to be disease-associated versus the fraction of positions having all variations predicted to be disease-associated ≥ the given threshold value.

We next examine the relationship between a protein being disease-associated and the fraction of “hot” spots in the protein of interest. Fig 4 shows the cumulative fraction of disease-associated proteins versus the fraction of “hot” spots in the proteins ≥ given threshold value. For all proteins (threshold value 0%), the fraction of variations being disease-associated is ~44%. This fraction jumps up to 86% for proteins having ≥50% “hot” spots and increases to 100% for proteins having ≥70% “hot” spots. The Pearson Correlation Coefficient of the two quantities is 0.97. Thus, the predicted “hot” spots are meaningful and can guide further experimental investigations. All the predictions are available at http://cssb.biology.gatech.edu/entprise/.

### Application of ENTPRISE to exome analysis

**Fig 5:**
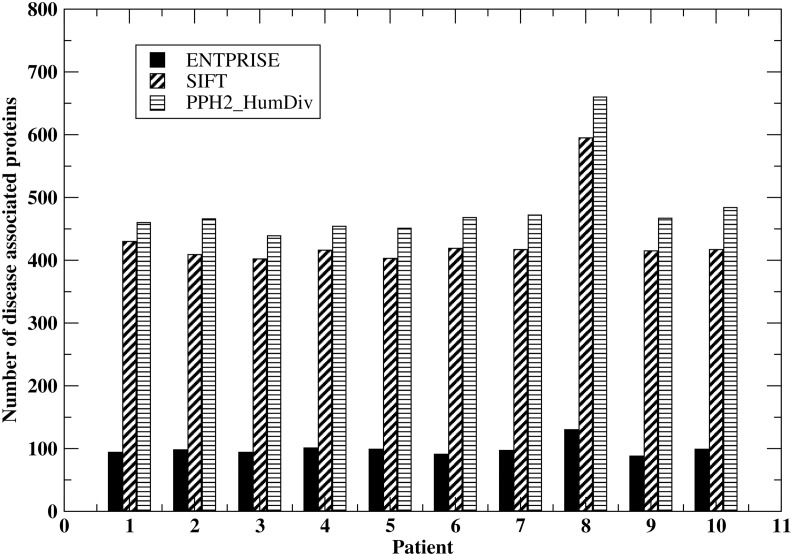
Number of disease-associated proteins predicted by ENTPRISE, SIFT, PPH2_HumDiv for variations from 10 patients.

We further demonstrate an advantage of ENTPRISE compared to the popular methods SIFT & PPH2 in a “real-life” application to human exomes. In practice, we filter the variations with allele frequency ≥ 0.17 in the 1k-genome database[2], i.e. any variation in a patient that also exists in the 1k-genome with allele frequency ≥ 0.17 will be assumed neutral. This is supported by the fact that, among the 17,830 variations with allele frequency ≥ 0.17 in the 1k-genome database, only 2 variations overlapped with disease-associated variations in our whole dataset ENTPRISE-TR+ENTPRISE-TE. Thus, the likelihood of a 1k-genome variation with allele frequency ≥ 0.17 being disease-associated is negligibly small (~0.01%). The filter will reduce the number of variations needed for further annotation to ~ ¼ because variations with large allele frequency are common in both healthy people and patients. We then apply ENTPRISE, SIFT & PPH2_HumDiv to the remaining variations of 10 patient cases (obtained from private communication). We compare how many proteins will be predicted by ENTPRISE, SIFT and PPH2_HumDiv to have disease-associated variations in Fig 5. Since SIFT, PPH2_HumDiv have slightly larger coverage than ENTPRISE, we report only their overlapped annotations with ENTPRISE. For all patients, ENTPRISE predicts around 100 proteins having disease-associated variations per exome whereas SIFT & PPH2_HumDiv predict ~ 400–500, *viz*. 4–5 times that of ENTPRISE. Thus, due to its low false positive rate (see Tables 4 and 6), ENTPRISE significantly reduces the number of disease-associated candidate proteins that might require further experimental or literature investigation. This is consistent with a strategy that minimizes the number of false positives at the expense of slightly lower coverage of the true positives.

### ENTPRISE web server

To facilitate fast, high throughput analysis of massive whole-exome sequencing data, we implemented the ENTPRISE webserver at http://cssb.biology.gatech.edu/entprise/. Since ENTPRISE requires protein structure predictions that are computationally expensive, the web service will only return results for variations having pre-computed protein structures for which all variation scores are pre-computed. The inputs to the server are the NCBI protein sequence ID, position of the amino acid substitution, the reference amino acid type and the substitution residue type. The server usually returns results in a few seconds. The output has ENTPRISE scores and cutoff information for disease-associated classification as well as sequence entropy for user’s reference. We have pre-computed scores for 33,093 of 33,610 Human proteins with an average coverage of 91.5% of the protein’s residues. These data can also be downloaded for local usage if necessary.

## Discussion

This work demonstrates the first comprehensive combination of sequence conservation and 3D structure models for the prediction of disease-associated variations in proteins. The PPH2 methods also utilize sequence & experimentally determined 3D structures from the PDB. However, PDB structures only cover less than one third of the human proteome[58]. As anticipated by our recent comparative study on the 1000 Genome variations and disease-associated variations[31], three dimensional structure modeling indeed significantly improves the performance of the current method. The recent work of FATHMM [6] that systematically incorporates domain specific weights (similar to the domain composition based on structure term that is included in this work) performs well on conventional testing datasets as compared to its own unweighted version and other methods (Table 4)[6]. It also performs much better than other methods in terms of the false positive rate (Table 6). Here, we show a consistent result that including domain composition significantly lowers the false positive rate. ENTPRISE’s machine learning approach makes the effects of domain composition even more pronounced for lowering the false positive rate than FATHMM (see also ENTPRISE_ONLYDOM’s performance). However, inclusion of only domain level structure information is not good enough for discriminating disease-associated variations from neutral ones on the same gene/protein as seen in the rather poor performance of FATHMM on the ENTPRISE-balance set (Table 5). The inclusion of sequence conservation, amino acid type and contacting residue composition is needed for the improved ability of current method to discriminate disease-associated variations from neutral ones within a gene/protein. Thus, the combination of the features (sequence entropy, amino acid type, domain composition, contacting residue composition) makes ENTPRISE perform well in all tests.

A recent method called SuSPect by Yates et.al. [59] utilizes protein level propensity for being disease-associated by integrating interaction network information through a Support Vector Machine (SVM) approach [45]. It also investigated the utility of modeled protein structures in predicting disease-associated variations, and concluded, contrary to this work, that structure information does not improve predictive ability. However, the conclusion is based on performance on the conventional VariBench[60], that consists of mainly proteins having variations dominated either by neutral or by disease-associated ones. Thus, SuSPect has the same issue as FATHMM in that it performs well on unbalanced datasets like VariBench & PredictSNP [6, 17, 60], but does not perform as well on a balanced set like ENTPRISE-balance constructed in this work. Indeed, an evaluation of SuSPect on the ENTPRISE-balance set shows that its MCC and accuracy are 0.348 and 0.665, respectively. This performance is better than FATHMM because of its inclusion of sequence conservation, but it is still very similar to those of other sequence conservation dominated methods. We also note that there is a performance discrepancy of FATHMM in the SuSPect work[59] from that in the original FATHMM’s report[6] for the VariBench dataset. In fact, for the subset of VariBench evaluated in SuSPect work[59], we find a MCC value of 0.72 by FATHMM, that is the same as that given in the FATHMM’s work[6] for the whole VariBench dataset. This is better than SuSPect’s best value 0.65. For the same subset (overlaps with our training set ENTPRISE-TR were excluded), ENTPRISE has a MCC value of 0.78 that is much better than SuSPect’s best value of 0.65 and those of all other methods compared in SuSPect work[59]. This result and the 10 fold family level cross-validation (see S2 File) demonstrate that ENTPRISE has robust performance across diverse datasets.

Besides outstanding performance compared to several other state-of-the-art methods, ENTPRISE also provides the 3D structures for manual inspection and interpretation of the predictions. Specific structural features, e.g., if a variation is within a ligand binding pocket, protein-protein binding interface, enzyme activity site, disulfide bond, H-bond, will further assist in filtering variations. Because these features are not always available for all variations, they are currently not integrated into ENTPRISE to assess disease association but rather are evaluated after ENTPRISE is run to ascertain what the possible functional consequence of the variation might be. We are currently working on improving predictions of binding sites, interface sites, enzyme activity sites on the predicted protein structures. These will be utilized for further improvements of ENTPRISE.

Another strength of ENTPRISE is that it also performs well and better than some of the established methods such as MUTATIONASSESSOR and PolyPhen-2 for cancer driver prediction on the COSMIC data. The consistency of ENTPRISE’s performance on Mendelian diseases and cancers indicates that somatic variations are not special relative to genetic variations but rather they share common features.

Predictions of ENTPRISE on human nucleic acid binding proteins show that some of them are very “hot” in that most positions are likely disease-associated, e.g. the TP53 protein, while many other proteins only have a tiny fraction (<5%) of “hot” spots that are disease-associated. The fraction of “hot” spots is highly correlated with the protein being disease-associated, which in turn is highly correlated with the number of protein-protein interactions it experiences.

A slight disadvantage of ENTPRISE as with the FATHMM method is that it does not provide 100% coverage of a given exome. A few large proteins (1.5% or 517, out of 33,610 human exome proteins) cannot be modeled. Some proteins also have regions that cannot be reliably modeled (on average 8.5% of the residues in a protein lie in regions that we cannot model at the moment). Thus, its expected coverage is around 90%. Nevertheless, this small reduction in coverage should not impair its utility in practical applications. This is especially true in real world exome applications, where the number of putative disease-associated proteins is roughly a factor of 5 less than either SIFT or PPH2 (these two have much higher false positive rates). Thus, it is a useful tool for assisting in the examination of clinical exomes.

It should also be noted that even though ENTPRISE can predict disease associated/cancer driver variations, it does not necessarily mean that it can predict loss or gain of biochemical function (though anecdotal analysis suggests that this is often the case). Moreover, even if it could, there is no guarantee that such a functional change will cause the disease of interest. Only changes in proteins that play an essential physiological role underlying a disease are contributory to the disease. In our assumption that the 1000 Genome data to be neutral, the word “neutral” should not be taken as functionally neutral, but disease-associated neutral.

## Supporting Information

S1 Fig Fraction of human proteome targets with at least one domain having a predicted TM-score ≥ given the value. (TIF)

S2 Fig Receiver operating characteristic curve of ENTPRISE for 10 fold cross-validation test on ENTPRISE-TR+ENTPRISE-TE set. Shown in figure are the combined predictions of the 10 fold cross-validation.
(TIF)

S3 Fig Histogram of the number of sequence homologs for 1,461 proteins with both disease associated & neutral variations; and 8,264 proteins with only neutral variations in the ENTPRISE-TE set. (TIF)

S1 File ENTPRISE data. ENTPRISE datasets and prediction results. (ZIP)

S2 File Additional test results for ENTPRISE. (PDF)

S1 Table 10-fold cross-validation test of ENTPRISE. (DOCX)

S2 Table Performance of SVM vs Boosted Tree based ENTPRISE. (DOCX)

S3 Table Performance of ENTPRISE for proteins with few homologs. (DOCX)

S4 Table Dependence of ENTPRISE on training data. (DOCX)

S5 Table Performance of methods on variants not predicted by ENTPRISE and vice versa. (DOCX)
