# Supplementary figures and images for: ENTPRISE: An Algorithm for Predicting Human Disease-Associated Amino Acid Substitutions from Sequence Entropy and Predicted Protein Structures
Source: PLoS One. 2016 Mar 16;11(3):e0150965. doi: 10.1371/journal.pone.0150965 (PMC4794227; doi:10.1371/journal.pone.0150965)

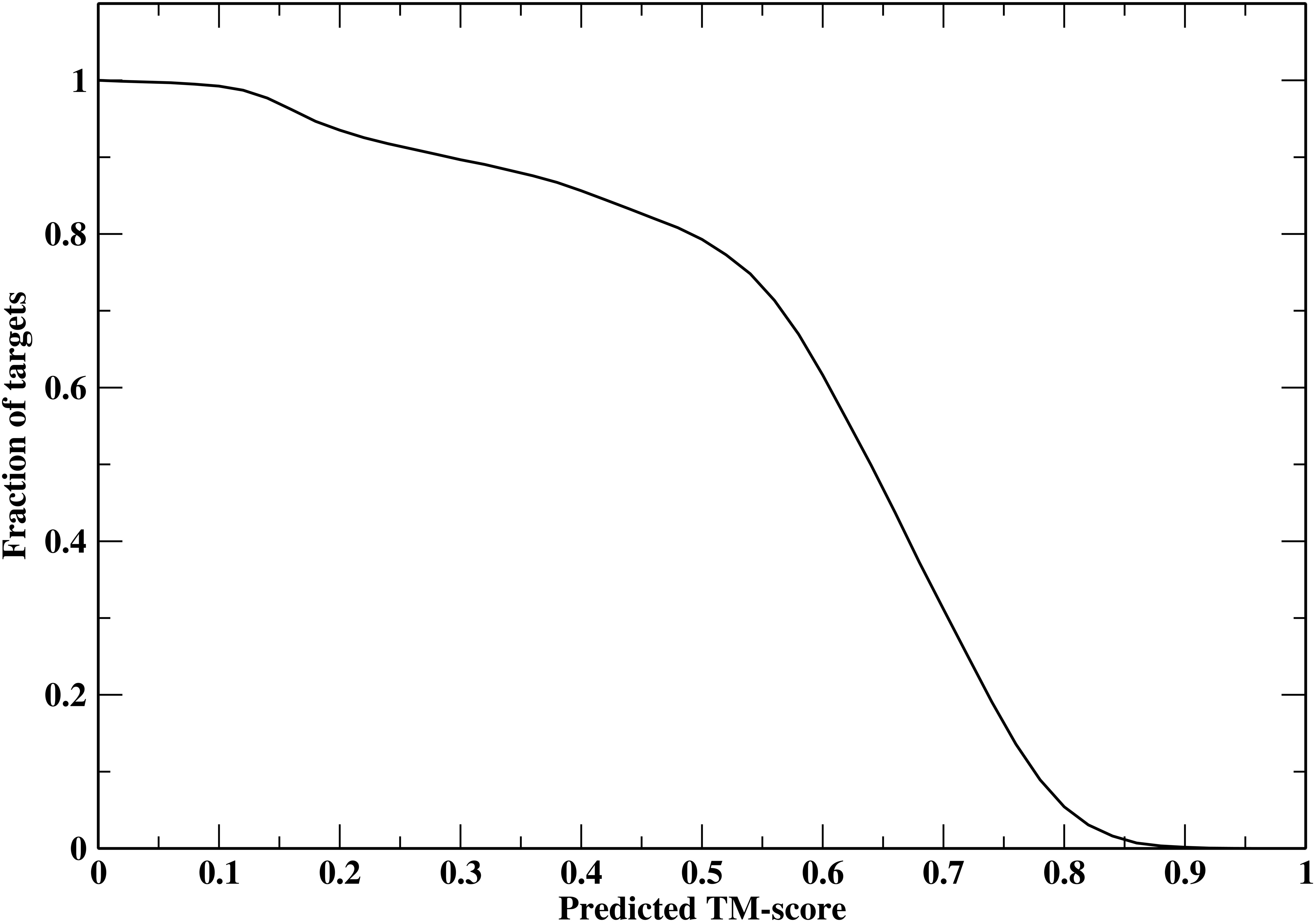

Supplement: S1 Fig — (TIF) [file pone.0150965.s001.tif]

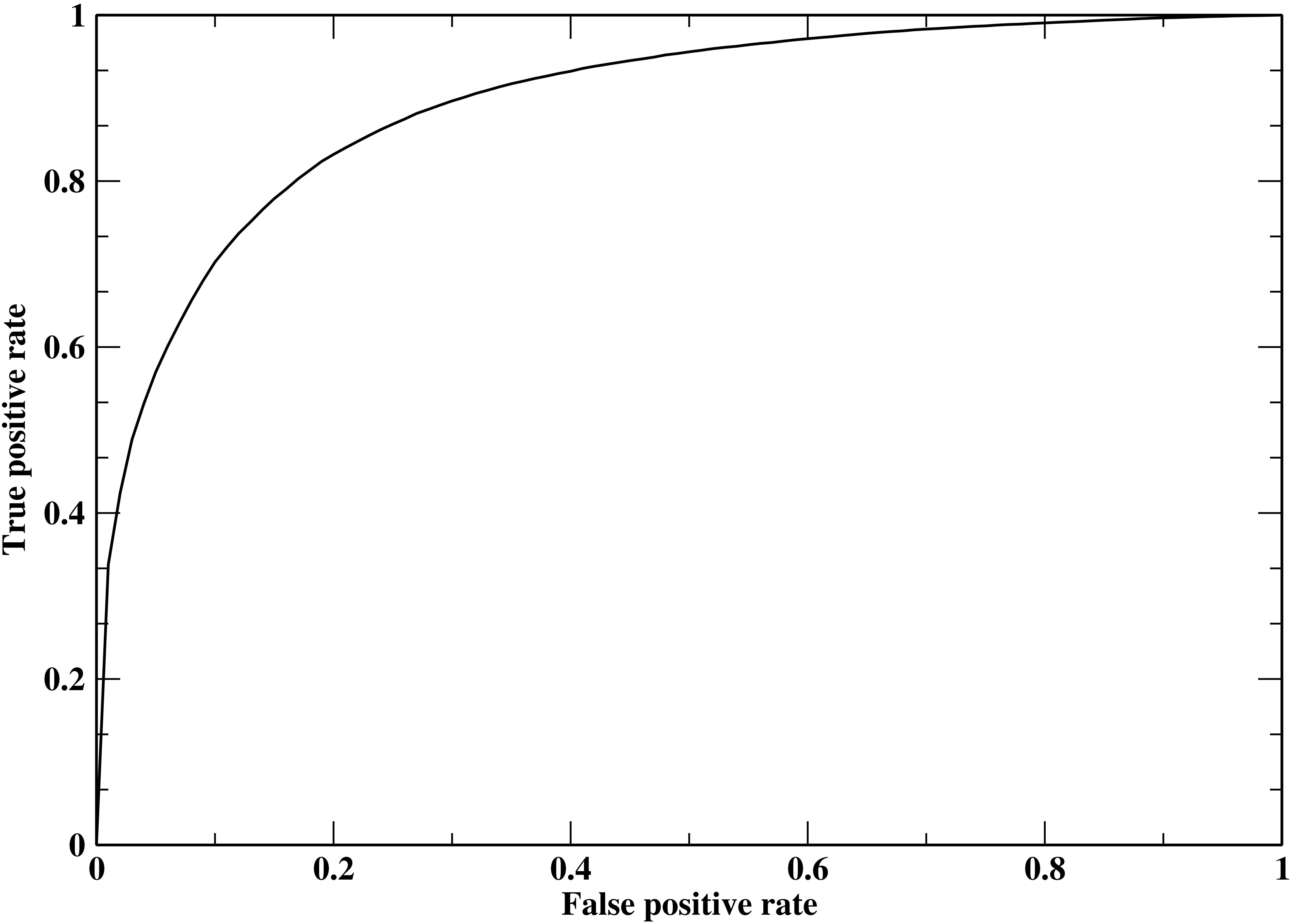

Supplement: S2 Fig — Shown in figure are the combined predictions of the 10 fold cross-validation. (TIF) [file pone.0150965.s002.tif]

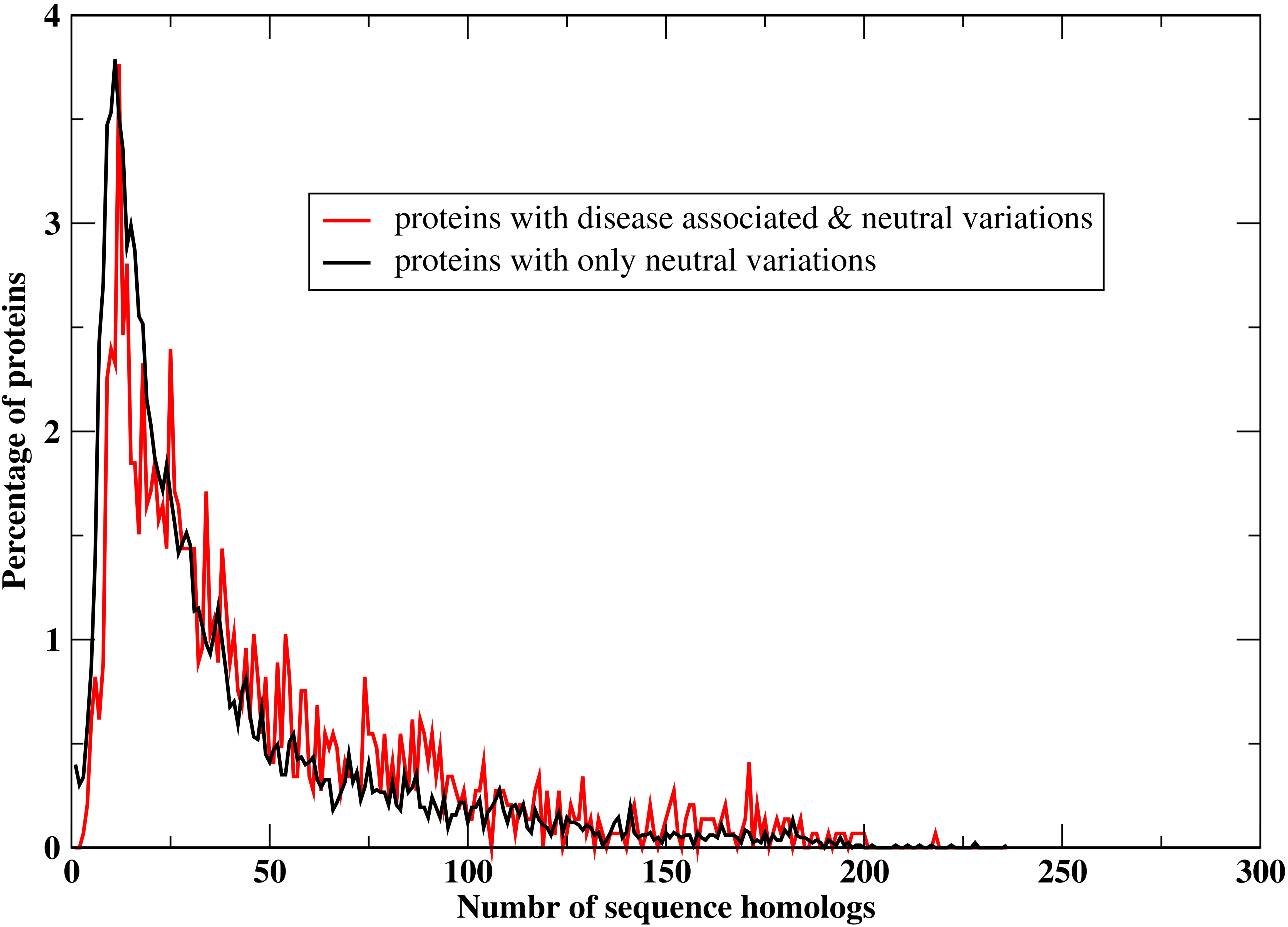

Supplement: S3 Fig — (TIF) [file pone.0150965.s003.tif]
