# Supplementary material for: ENTPRISE: An Algorithm for Predicting Human Disease-Associated Amino Acid Substitutions from Sequence Entropy and Predicted Protein Structures
Source: PLoS One. 2016 Mar 16;11(3):e0150965. doi: 10.1371/journal.pone.0150965 (PMC4794227; doi:10.1371/journal.pone.0150965)
Supplement: S2 File — (PDF) [file pone.0150965.s005.pdf]

Supplementary Information for

ENTPRISE: An algorithm for predicting human disease-associated amino acid substitutions from sequence entropy and predicted protein structures

by

Hongyi Zhou, Mu Gao and Jeffrey Skolnick*

Center for the Study of Systems Biology, School of Biology, Georgia Institute of Technology, 250 14th Street, N.W., Atlanta, GA 30318

*To whom all correspondence should be addressed.

TEL: 404-407-8975

FAX: 404-385-7478

E-mail: skolnick@gatech.edu

Model quality of Human proteome targets

The cumulative distribution of model quality as assessed by the predicted TM-score derived from TASSER simulation for the Human proteome is given in S1 Fig. The total number of modeled targets is 32,579. The average predicted TM-score is 0.60±0.18 and median predicted TM-score is 0.64.

Ten-fold Cross-validation

To ensure that ENTPRISE is not biased towards a particular training set and has a consistent predictive capability for family/domain not seen in the training set, we performed an additional 10-fold cross-validation test at disjoint family/domain level instead of the two fold variation level partition described in main text. Protein sequences are mapped to PFAM family/domains (1). We then randomly partition all the distinct family/domains into 10 disjoint (non-overlapping) subsets with approximately equal numbers of variations. For each family/domain subset, their corresponding amino acid variations in the combined ENTPRISE-TR+ENTPRISE-TE set were selected as the testing subsets. Variations of ENTPRISE-TR+ENTPRISE-TE within the other 9 subsets were utilized for training. To test if the default number of tree (N_{tree} = 2000) makes ENTPRISE susceptible to overtraining, we also perform a similar test for ENTPRISE with various smaller tree numbers (N_{tree}=500,1000,1500). Results are compiled in S1 Table. The average MCC of ENTPRISE on the whole set is 0.571 ±0.020, which is much smaller than 0.645 obtained in the two-fold test result listed in Table 4. However, this is the performance of ENTPRISE under the hardest possible situation and MCC=0.571 is still better than those of other methods. Small standard deviations indicate that

ENTPRISE has consistent performance regardless of training/testing partition. The ROC curves of ENTPRISE in 10 fold cross validation is shown in S2 Fig. At a 10% (20%) false positive rate, the true positive rate is 70.2% (83.2%).

Performance of SVM

We tested the use of a support vector machine (SVM) (2) for regression instead of boosted tree in ENTPRISE. Here, the SVM-light implementation with radial basis kernel function and default parameters was used (2); all features are exactly the same as those used in ENTPRISE. The results are compiled in S2 Table along with those of ENTPRISE. They clearly show that the boosted tree regression in ENTPRISE is superior to an SVM based on overall measures such as MCC, OPM, AUC, and false positive rate.

Histogram of number of sequence homologs for proteins in the ENTPRISE-TE set

By using an E-value cutoff of 0.001 and 5 iterations of PSIBLAST searching the non-redundant (NR) sequence database downloaded from the Protein Data Bank, we obtained the homologous sequences of each of the 9,725 proteins in the ENTPRISE-TE test set. The histogram of the number of sequence homologs is given in S3 Fig. The number of sequence homologs range from 1 to 236 with a peak at around 11, and an average (median) number of 39 (24). The distributions of proteins with both disease and neutral variations and proteins with only neutral variations are similar. The performance of ENTPRISE on 3,505 variants on 1,114 proteins having few or none homologs is given in S3 Table. The MCC value of 0.554 for this subset is worse than that for whole ENTPRISE-TE set 0.645 indicating that the number of homologs only slightly affects the performance of ENTPRISE.

Performance of methods on variants not predicted by ENTPRISE and vice versa

There are 6,632 variants in the training plus testing data sets of PredictSNP methods not predicted by ENTPRISE and thus are not included in the ENTPRISE-TE and ENTPRISE-balance sets. ENTPRISE has no prediction for 23,070 (12.4% of total selected variants) variants in the 1000 Genome set. For those variants, we compile the results by other methods in S5 Table along with results by ENTPRISE on those not predicted by other methods. For ENTPRISE-TE set, ENTPRISE has the best overall performance based on OPM and AUC, and FATHMM is the best based on the MCC. For the ENTPRISE-balance set, ENTPRISE has the best AUC value of 0.776, and PPH2_HVAR has the best MCC value of 0.412. For the 1000 Genomes set, ENTPRISE still has the lowest false positive rate of 8.7% whereas other methods have at least around twice the false positive rate.

REFERENCES

1. Finn, R., Tate, J., Mistry, J., Coggill, P., Sammut, S., Hotz, H., Ceric, G., Forslund, K., Eddy, S., Sonnhammer, E. *et al.* (2009) The Pfam protein families database. *Nucl. Acid. Res.*, **36**, D281--288.
2. Joachims, T. (1998) In Scholkopf, B., Burges, C. and Smola, A. (eds.), *Advances in Kernel Methods - Support Vector Learning*. MIT Press, Cambridge, USA.
