# Supplementary material for: ENTPRISE: An Algorithm for Predicting Human Disease-Associated Amino Acid Substitutions from Sequence Entropy and Predicted Protein Structures
Source: PLoS One. 2016 Mar 16;11(3):e0150965. doi: 10.1371/journal.pone.0150965 (PMC4794227; doi:10.1371/journal.pone.0150965)
Supplement: S1 Table — (DOCX) [file pone.0150965.s006.docx]

**Table S1**

**10-fold cross-validation test of ENTPRISE** ^a^

| **Method** | **MCC** | **ACC** | **Sen** | **Spe** | **PPV** | **NPV** | **AUC** |
| --- | --- | --- | --- | --- | --- | --- | --- |
| ENTPRISE | 0.571  ±0.020 | 0.818  ±0.006 | 0.701  ±0.030 | 0.870  ±0.010 | 0.705  ±0.030 | 0.866  ±0.015 | 0.874  ±0.008 |
|  |  |  |  |  |  |  |  |
| ENTPRISE(N_tree_=500) | 0.486  ±0.013 | 0.773  ±0.010 | 0.686  ±0.030 | 0.813  ±0.019 | 0.622  ±0.033 | 0.852  ±0.025 | 0.827  ±0.005 |
| ENTPRISE(N_tree_=1000) | 0.525  ±0.013 | 0.790  ±0.005 | 0.721  ±0.014 | 0.821  ±0.010 | 0.642  ±0.033 | 0.867  ±0.016 | 0.850  ±0.005 |
| ENTPRISE(N_tree_=1500) | 0.553  ±0.018 | 0.808  ±0.005 | 0.701  ±0.025 | 0.856  ±0.011 | 0.685  ±0.034 | 0.865  ±0.015 | 0.866  ±0.007 |

^a^ For each method, shown in table are the average & standard deviation of the 10 test subsets, each assessed independently.
