# Supplementary material for: ENTPRISE: An Algorithm for Predicting Human Disease-Associated Amino Acid Substitutions from Sequence Entropy and Predicted Protein Structures
Source: PLoS One. 2016 Mar 16;11(3):e0150965. doi: 10.1371/journal.pone.0150965 (PMC4794227; doi:10.1371/journal.pone.0150965)
Supplement: S2 Table — (DOCX) [file pone.0150965.s007.docx]

**Table S2**

**Performance of SVM vs Boosted Tree based ENTPRISE**

| **ENTPRISE-TE set** | | | | | | | | | | |
| --- | --- | --- | --- | --- | --- | --- | --- | --- | --- | --- |
| **Method** | **Evaluated**  **variations** | **MCC** | **ACC** | **Sen** | | **Spe** | **PPV** | **NPV** | **OPM** | **AUC** |
| ENTPRISE | 46574 | **0.645** | **0.847** | 0.768 | | **0.883** | **0.746** | **0.894** | **0.565** | **0.907** |
| SVM | 46574 | 0.376 | 0.669 | **0.794** | | 0.613 | 0.480 | 0.869 | 0.322 | 0.779 |
|  | | | | | | | | | | |
| **ENTPRISE-balance set** | | | | | | | | | | |
| ENTPRISE | 8907 | **0.493** | **0.742** | 0.669 | | **0.819** | **0.790** | 0.708 | **0.415** | **0.818** |
| SVM | 8907 | 0.404 | 0.699 | **0.802** | | 0.593 | 0.668 | **0.746** | 0.345 | 0.773 |
|  | | | | | | | | | | |
| **1000 Genome & VariSNP sets** | | | | | | | | | | |
|  | **1000 Genome** | | | | **VariSNP** | | | | | |
|  | **Evaluated**  **variations** | **False**  **positive rate** | | | **Evaluated**  **variations** | | | **False**  **positive rate** | | |
| ENTPRISE | 162,249 | **10.7%** | | | 61,215 | | | **9.0%** | | |
| SVM | 162,249 | 40.3% | | | 61,215 | | | 37.8% | | |
