# Supplementary material for: ENTPRISE: An Algorithm for Predicting Human Disease-Associated Amino Acid Substitutions from Sequence Entropy and Predicted Protein Structures
Source: PLoS One. 2016 Mar 16;11(3):e0150965. doi: 10.1371/journal.pone.0150965 (PMC4794227; doi:10.1371/journal.pone.0150965)
Supplement: S3 Table — (DOCX) [file pone.0150965.s008.docx]

Table S3

**Performance of ENTPRISE for proteins with few homologs**

| **Evaluated**  **variations** | **MCC** | **ACC** | **Sen** | **Spe** | **PPV** | **NPV** | **OPM** | **AUC** |
| --- | --- | --- | --- | --- | --- | --- | --- | --- |
| 3,505 | 0.554 | 0.896 | 0.601 | 0.942 | 0.627 | 0.937 | 0.565 | 0.883 |
