# Supplementary material for: ENTPRISE: An Algorithm for Predicting Human Disease-Associated Amino Acid Substitutions from Sequence Entropy and Predicted Protein Structures
Source: PLoS One. 2016 Mar 16;11(3):e0150965. doi: 10.1371/journal.pone.0150965 (PMC4794227; doi:10.1371/journal.pone.0150965)
Supplement: S4 Table — (DOCX) [file pone.0150965.s009.docx]

**Table S4**

**Dependence of ENTPRISE on training data**

| **ENTPRISE-TE set** | | | | | | | | |
| --- | --- | --- | --- | --- | --- | --- | --- | --- |
| Training data | MCC | ACC | Sen | Spe | PPV | NPV | OPM | AUC |
| Observed data | 0.713 | 0.878 | 0.794 | 0.916 | 0.809 | 0.908 | 0.636 | 0.929 |
| Balanced data (default) | 0.645 | 0.847 | 0.768 | 0.883 | 0.746 | 0.894 | 0.565 | 0.907 |
|  | | | | | | | | |
| **ENTPRISE-balance set** | | | | | | | | |
| Observed data | 0.480 | 0.733 | 0.621 | 0.847 | 0.806 | 0.686 | 0.404 | 0.813 |
| Balanced data (default) | 0.493 | 0.742 | 0.669 | 0.819 | 0.790 | 0.708 | 0.415 | 0.818 |
|  | | | | | | | | |
| **1000 Genome & VariSNP sets** | | | | | | | | |
|  | **1000 Genome** | | | | **VariSNP** | | | |
|  | False positive rate | | | | False positive rate | | | |
| Observed data | 6.8% | | | | 5.6% | | | |
| Balanced data (default) | 10.7% | | | | 9.0% | | | |
