# Supplementary material for: ENTPRISE: An Algorithm for Predicting Human Disease-Associated Amino Acid Substitutions from Sequence Entropy and Predicted Protein Structures
Source: PLoS One. 2016 Mar 16;11(3):e0150965. doi: 10.1371/journal.pone.0150965 (PMC4794227; doi:10.1371/journal.pone.0150965)
Supplement: S5 Table — (DOCX) [file pone.0150965.s010.docx]

**Table S5**

**Performance of methods on variants not predicted by ENTPRISE and vise versa**

| **ENTPRISE-TE set** | | | | | | | | | |
| --- | --- | --- | --- | --- | --- | --- | --- | --- | --- |
| **Method** | **Evaluated**  **variations** | **MCC** | **ACC** | **Sen** | **Spe** | **PPV** | **NPV** | **OPM** | **AUC** |
| ENTPRISE**^a^** | 3,524 | 0.490 | 0.886 | 0.712 | 0.903 | 0.421 | 0.970 | **0.458** | **0.896** |
| SIFT | 5,434 | 0.396 | 0.687 | 0.791 | 0.643 | 0.480 | 0.881 | 0.338 | 0.801 |
| PPH2_HDIV | 5,442 | 0.414 | 0.687 | 0.824 | 0.630 | 0.481 | 0.896 | 0.349 | 0.811 |
| PPH2_HVAR | 5,442 | 0.491 | 0.757 | 0.784 | 0.746 | 0.562 | 0.892 | 0.418 | 0.838 |
| MUTATIONASSESSOR | 5,377 | 0.454 | 0.761 | 0.678 | 0.795 | 0.581 | 0.855 | 0.394 | 0.808 |
| MUTATIONTASTER | 5,438 | 0.405 | 0.654 | 0.881 | 0.559 | 0.454 | 0.919 | 0.335 | 0.749 |
| FATHMM | 5,386 | **0.531** | 0.745 | 0.904 | 0.678 | 0.541 | 0.944 | 0.443 | 0.855 |
| **ENTPRISE-balance set** | | | | | | | | | |
| ENTPRISE | 178 | 0.385 | 0.691 | 0.598 | 0.780 | 0.722 | 0.670 | 0.332 | **0.776** |
| SIFT | 993 | 0.402 | 0.708 | 0.795 | 0.598 | 0.715 | 0.697 | 0.346 | 0.774 |
| PPH2_HDIV | 1,001 | 0.371 | 0.693 | 0.793 | 0.567 | 0.699 | 0.683 | 0.324 | 0.755 |
| PPH2_HVAR | 1,001 | **0.412** | 0.708 | 0.718 | 0.696 | 0.750 | 0.660 | **0.353** | 0.767 |
| MUTATIONASSESSOR | 990 | 0.388 | 0.692 | 0.665 | 0.726 | 0.751 | 0.635 | 0.334 | 0.751 |
| MUTATIONTASTER | 997 | 0.313 | 0.666 | 0.831 | 0.457 | 0.660 | 0.681 | 0.286 | 0.674 |
| FATHMM | 993 | 0.153 | 0.596 | 0.856 | 0.267 | 0.597 | 0.594 | 0.196 | 0.628 |
| **1000 Genome set** | | | | | | | | | |
|  | Evaluated variations | | | | False positive rate | | | | |
| ENTPRISE | 15,281 | | | | **8.7%** | | | | |
| SIFT | 19,928 | | | | 41.1% | | | | |
| PPH2_HDIV | 19,989 | | | | 47.0% | | | | |
| PPH2_HVAR | 19,989 | | | | 34.5% | | | | |
| MUTATIONASSESSOR | 19,675 | | | | 26.0% | | | | |
| MUTATIONTASTER | 19,968 | | | | 51.6% | | | | |
| FATHMM | 19,462 | | | | 15.7% | | | | |

^a^ENTPRISE predictions are for those not predicted by other methods.
